# Accumulation of cytoplasmic Cdk1 is associated with cancer growth and survival rate in epithelial ovarian cancer

**DOI:** 10.18632/oncotarget.10373

**Published:** 2016-07-01

**Authors:** Wookyeom Yang, Hanbyoul Cho, Ha-Yeon Shin, Joon-Yong Chung, Eun Suk Kang, Eun-ju Lee, Jae-Hoon Kim

**Affiliations:** ^1^ Department of Obstetrics and Gynecology, Gangnam Severance Hospital, Yonsei University College of Medicine, Seoul, Korea; ^2^ Institute of Women's Life Medical Science, Yonsei University College of Medicine, Seoul, Korea; ^3^ Tissue Array Research Program, Laboratory of Pathology, National Cancer Institute, National Institutes of Health, Bethesda, MD, USA; ^4^ Department of Laboratory Medicine, Samsung Medical Center, Sungkyunkwan University School of Medicine, Seoul, Korea

**Keywords:** Cdk1, cytoplasmic accumulation, epithelial ovarian cancer, RO-3306

## Abstract

Cyclin dependent kinase 1 (Cdk1) have previously reported correlation with cancer growth and a key regulator for cell cycle. Mostly, Cdk1′s function of nucleus for cell cycle is well known to be associated with cancer, but cytoplasmic Cdk1′s traits are not clearly identified, yet. We revealed that tissue microarray blocks of epithelial ovarian cancer (*n* = 249) showed increased level of cytoplasmic Cdk1 (*p* < 0.001), but not in nucleus (*p* = 0.192) of histologic cell type independently. On survival analysis, Cdk1 overexpression conferred a significantly worse prognosis in 5-year overall survival (Log-rank *p* = 0.028, Hazard ratio = 2.016, 95% CI = 1.097 to 4.635). Also, the expression of Cdk1 was increased in ovarian cancer cell lines and Gene Expression Omnibus datasets. When the expression and activity of Cdk1 were inhibited by si-Cdk1 or RO-3306 which is a potent Cdk1 inhibitor, the growth of ovarian cancer was diminished. Moreover, combined treatment with RO-3306 and cisplatin in ovarian cancer significantly elevated anti-cancer effects than single-agent treatment. In conclusion, cytoplasmic Cdk1 expression which was elevated in ovarian cancer predicts a poor overall survival. The inhibition of Cdk1 expression and activity reduced ovarian cancer growth.

## INTRODUCTION

Cyclin-dependent kinases (CDKs) are important cell cycle-regulating proteins, which belong to a serine/threonine kinase family that comprise of a catalytic kinase subunit, together with cyclin protein partners. There are at least 13 different CDKs and more than 25 cyclin proteins identified to date [1]. However, only Cdk1, 2, 4, and 6 are directly involved in the cell cycle, and of these, Cdk2, 4, and 6 are not essential to the cell cycle. Furthermore, unlike other Cdks, Cdk1 can promote the cell cycle alone and is essential for cell cycle progression and cell division [1–3]. Cdk1 regulates the G2 phase as part of a complex with cyclin A and is involved in G2/M transition by forming a complex with cyclin B. Knockdown of Cdk1 increases G2/M arrest during the cell cycle and results in polyploid cells [1, 2, 4].

Cdk1 is highly expressed in various cancers. It has been reported that Cdk1 expression or activity is elevated in Hodgkin's lymphomas [5], human colorectal cancer [6], prostate cancer [7], gastric lymphoma [8], childhood acute lymphoblastic leukemia [9] Therefore, Cdk1 is closely related with cancer progression. In particular, it has been reported that prognosis is poor in colorectal cancer patients with a high nuclear/cytoplasmic ratio of Cdk1 [6]. When activity of CDK1 and CDK2 was high in renal cell carcinoma patients, recurrence prediction rate was also high, while progression-free survival rate was low [10]. Therefore, Cdk1 has been introduced as a promising gene for targeted therapy that could inhibit progress of the cell cycle in various cancers and induce apoptosis. A number of Cdk inhibitors have been developed to date and their effects have also been studied. Inhibition of Cdk1 and Cdk2 by 6,7,4′-trihydroxyisoflavone inhibited growth of HCT-116, a human colon cancer cell line [11], and JNJ-7706621, an inhibitor of Cdk1/cyclinB1 and aurora kinase effectively reduced growth of transplantable liver tumor in combination treatment with paclitaxel [12]. Knockdown of cdc2 in glioblastoma U87 and U251 raised cell sensitivity to anticancer drugs such as temozolomide, resulting in reduction of cell proliferation and promotion of apoptosis [13].

In general, the cell cycle-associated activity of Cdk1 is known to occur in the nucleus. Although previous reports suggest that Cdk1 has activity in the cytoplasm that is independent of its activity in the nucleus [14, 15], its precise mechanism of function and downstream targets are not well understood. Moreover, its association with ovarian cancer is also not clear.

In this study, we found interesting results that the expression of cytoplasmic cdk1 increased in ovarian cancer. And also, accumulated cytoplasmic Cdk1 is associated with 5-year overall survival in ovarian cancer patients. In addition, Cdk1 knockdown or treatment of Cdk1 inhibitor resulted in inhibition of cell growth via G2/M arrest and apoptosis in ovarian cancer cell lines. Finally, these results suggested that cytoplasmic Cdk1 plays a crucial role of cancer growth in epithelial ovarian cancer.

## RESULTS

### Cdk1 protein is elevated and accumulated in the cytoplasm of epithelial ovarian cancer

**Figure 1:**
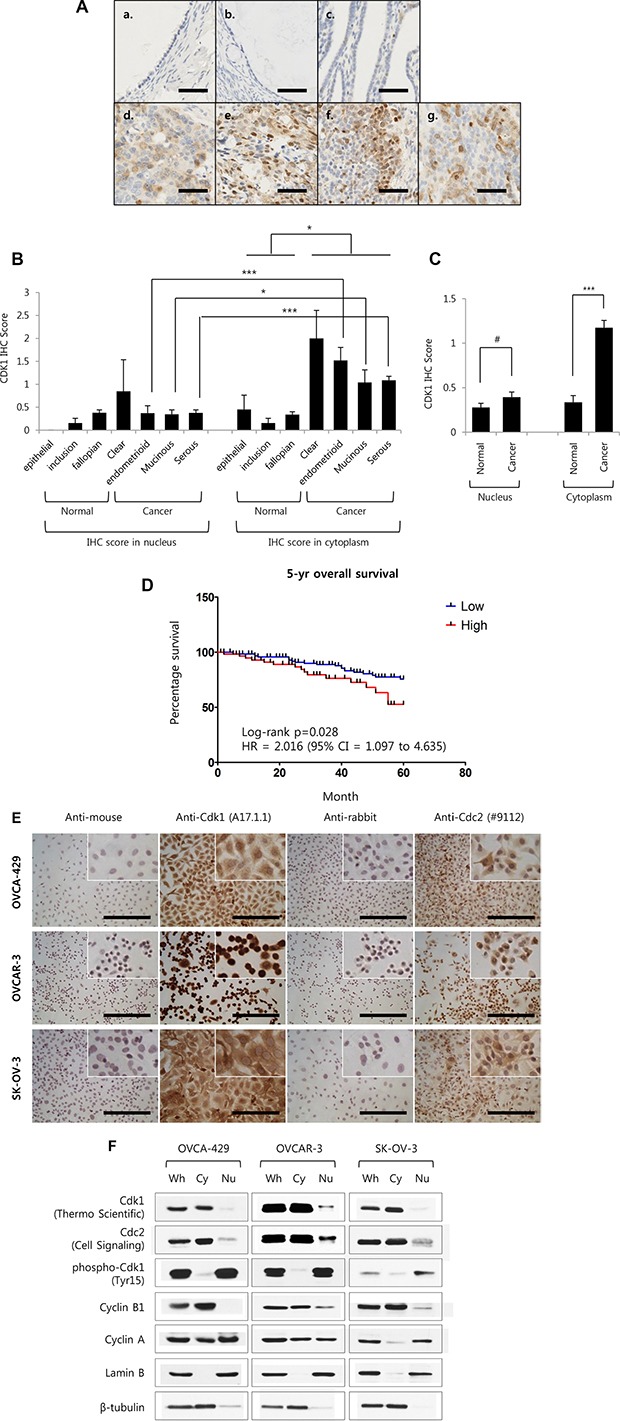
Cyclin dependent kinase 1 proteins in human ovarian cancer tissue specimens are accumulated in cytoplasm, and its expression is correlated with 5-yr survival rate (**A**) Representative immunohistochemical staining for Cdk1 in formalin-fixed, paraffin-embedded epithelial ovarian cancer tissues (EOC). (a, Epithelial; b, Inclusion cysts; c, Fallopian tube; d. Clear cell; e, Endometrioid; f, Mucinous; g, High-grade serous). Scale bar = 50 um. (**B**) IHC staining scores of Cdk1 in each indicated histology of EOC and Normal tissue samples. (Epithelial, *n* = 20; Inclusion cyst, *n* = 13; Fallopian tube, *n* = 71; Clear cell, *n* = 13; Endometrioid, *n* = 27; Mucinous, *n* = 26; Serous, *n* = 183). (**C**) Average IHC scores were combined with normal group (as epithelial, inclusion cyst, and fallopian tube; *n* = 104) and cancer group (as clear cell, endometrioid, mucinous, and serous; *n* = 249). Results are the means ± S.E. ***p < 0.001; *p < 0.05, ^#^*p* > 0.05. (**D**) Kaplan-Meier survival curve for patients with epithelial ovarian cancer was stratified according to cytoplasmic Cdk1 expression. (Low expression of cdk1 is 0 to 1 in IHC score, *n* = 128; High expression of cdk1 is more then 2, *n* = 61). (**E**) Representative immunocytochemical staining for Cdk1 in methanol-fixed, ovarian cancer cell lines (OVCA-429, OVCAR-3 and SK-OV-3). Scale bar = 100 um. (**F**) OVCA-429, OVCAR-3 and SK-OV-3 were performed subcellular fractionation from 70% density cultured cells and were analyzed via Western blot analysis using an anti-Cdk1 (Thermo Scientific's antibody), an anti-Cdc2 (Cell Signaling Technology's antibody), an anti-phospho-Cdk1 (Tyr15), an anti-Cyclin B1 and an anti-Cyclin A. Analysis of Lamin B (nuclear marker) and β-tubulin (cytoplasmic marker) was performed to assess the efficiency of subcellular fractionation. Whole cell lysate, Wh; Cytoplasm, Cy; Nuclear extract, Nu.

We previously performed microarray using five epithelial ovarian cancer (EOC) cell lines (YDOV-139, −157, −161, −13, and −151) that were established in the laboratory and four human ovarian surface epithelial (HOSE) cells [16–18]. Those microarray results were reanalyzed and showed that Cdk1 expression was induced in five YDOV cell lines (Fold change of Cdk1 expression; YDOV-13 = 3.16-fold, YDOV-139 = 7.83-fold, YDOV-151 = 2.39-fold, YDOV-157 = 3.63-fold, YDOV-161 = 2.10-fold). In order to determine if Cdk1 protein elevation shows clinicopathologic characteristics, immunohistochemistry (IHC) of ovarian cancer tissue samples was performed and classified by histology. Cytoplasmic Cdk1 expression was greater in cancer tissues than in normal tissues (Figure 1A–1C). The IHC scoring results are summarized in Table 1, which show that cytoplasmic Cdk1 expression significantly increased in all cell types including clear cell, endometrioid, mucinous, and serous types than in normal tissues, (*p < 0.05; ***p < 0.001) (Figure 1B and Table 1). When the normal tissue and cancer tissue groups were compared, cytoplasmic Cdk1 expression in the cancer tissue group was 3.44-fold than that in the normal tissue group (Figure 1C). In addition, there were 27 cytoplasm-stained tissue cores (26%), and 51 unstained tissue cores (49%) in normal tissues and 167 cytoplasm-stained tissue cores (67%) and 22 unstained tissue cores (9%) in cancer tissues (Table 2). Thus, while proportion of unstained tissues decreased in cancer tissues, proportion of cytoplasm-stained tissues increased. In addition, cytoplasmic Cdk1 expression increased in accordance with progression of tumor grade (*p* < 0.001) (Table 1). The prognosis of the high Cdk1-expression group was poor in terms of 5-year overall survival (log rank *p* = 0.028; hazard ratio [HR] = 2.016, 95% CI = 1.097 to 4.635) (Figure 1D). Patients with advanced FIGO stage, poor tumor grade, and serous type, showed significantly worse 5-yr overall survival (*p* = 0.0201, HR = 2.923 (95% CI = 1.146 to 4.827); *p* = 0.0038, HR = 2.984 (95% CI = 1.441 to 6.277); *p* = 0.0124, HR = 3.115 (95% CI = 1.209 to 4.722), respectively) than patients with early FIGO stage, well/moderate tumor grade, and non-serous type (Supplementary Figure S3). To verify Cdk1′s expression in ovarian cancer cell lines, in same results in tissue microarray, expression of Cdk1 was significantly detected more in cytoplasm via immunocytochemistry to utilize 3,3′-diaminobenzidine (DAB) staining (Figure 1E). To utilize western blot analysis after subcellular fractionation, the expression and activity of Cdk1 in ovarian cancer cell lines was strongly detected in cytoplasm (Figure 1F). Cyclin B1, known to interact with and regulate the activity of Cdk1, is mainly expressed in the cytoplasm of ovarian cancer cells. Cyclin A, although highly expressed in the nucleus, is also expressed in the cytoplasm. In addition, the significantly lower phosphorylation status of Tyr15, the Cdk1 inhibitory phosphorylation site [19], in the cytoplasm compared with that in the nucleus indicates that the cytoplasmic activity of Cdk1 is very high (Figure 1F). Therefore, it is possible that the high activity of cytoplasmic Cdk1 in ovarian cancer depends on cytoplasmic cyclins and reduced inhibitory phosphorylation.

**Table 1:** Cdk1 immunohistochemical staining score in EOC

	Number of Patients	Scores in nucleus				Scores in cytoplasm				*p* value (nucleus/cytoplasm)
		Mean	95% CI	Range	*p* value	Mean	95% CI	Range	*p* value	
**Histology**					0.192				< 0.001^*^	
Epithelial	20	0.00				0.45	(−0.20–1.10)	0–6		
Inclusion cyst	13	0.15	(−0.07–0.38)	0–1		0.15	(−0.07–0.38)	0–1		1.000
Fallopian tube	71	0.38	(0.26–0.50)	0–2		0.34	(0.21–0.46)	0–2		0.632
Clear cell	13	0.85	(−0.65–2.34)	0–9		2.00	(0.67–3.33)	0–9		0.221
Endometrioid	27	0.37	(0.04–0.70)	0–4		1.52	(0.93–2.10)	0–6		< 0.001^*^
Mucinous	26	0.35	(0.15–0.54)	0–1		1.04	(0.47–1.60)	0–6		0.021^*^
Serous	183	0.38	(0.25–0.50)	0–6		1.08	(0.91–1.25)	0–9		< 0.001^*^
**Diagnostic category**					0.233				< 0.001^*^	
Normal	104	0.28	(0.19–0.37)	0–2		0.34	(0.19–0.48)	0–6		0.511
Cancer	249	0.40	(0.28–0.51)	0–9		1.17	(1.02–1.33)	0–9		< 0.001^*^
**FIGO stage**					0.797				0.307	
I / II	57	0.47	(0.13–0.81)	0–9		1.39	(0.89–1.88)	0–9		0.003^*^
III / IV	168	0.38	(0.26–0.49)	0–6		1.10	(0.92–1.26)	0–6		< 0.001^*^
Recurrent	24	0.38	(−0.15–0.90)	0–6		1.29	(0.82–1.77)	0–4		0.010^*^
**Tumor grade**					0.123				< 0.001^*^	
1	16	0.81	(0.33–1.30)	0–3		0.63	(0.2–1.06)	0–2		0.542
2	73	0.22	(0.11–0.32)	0–2		1.08	(0.86–1.30)	0–4		< 0.001^*^
3	81	0.42	(0.14–0.70)	0–9		1.63	(1.24–2.00)	0–9		< 0.001^*^
x	79	0.46	(0.25–0.66)	0–6		0.91	(0.68–1.14)	0–4		0.004^*^
**Chemoresponse**					0.599				0.859	
Resistance	66	0.45	(0.17–0.74)	0–6		1.21	(0.91–1.51)	0–6		< 0.001^*^
sensitive	163	0.36	(0.22–0.49)	0–6		1.15	(0.94–1.36)	0–9		< 0.001^*^
x	20	0.55	(0.08–1.02)	0–4		1.30	(0.71–1.89)	0–4		0.044^*^

CI, confidence interval; FIGO, International Federation of Gynecology and Obsterics; x, undefined.

* *p* < 0.05.

**Table 2:** Number of Cdk1 stained cores in ovarian cancer TMA blocks

	Nucleus	Cytoplasm	Unstained	Total core
Normal	28 (27%)	27 (26%)	51 (49%)	104
Cancer	66 (27%)	167 (67%)	22 (9%)	249

Thus, as normal tissue progressed to cancer tissue, expression of Cdk1, particularly in the cytoplasm, increased considerably. And, that cytoplasmic Cdk1 expression is correlated with ovarian cancer patient's survival rate.

### Cdk1 and cyclinB1 are overexpressed in epithelial ovarian cancer comparing with human ovarian surface epithelial cells

**Figure 2:**
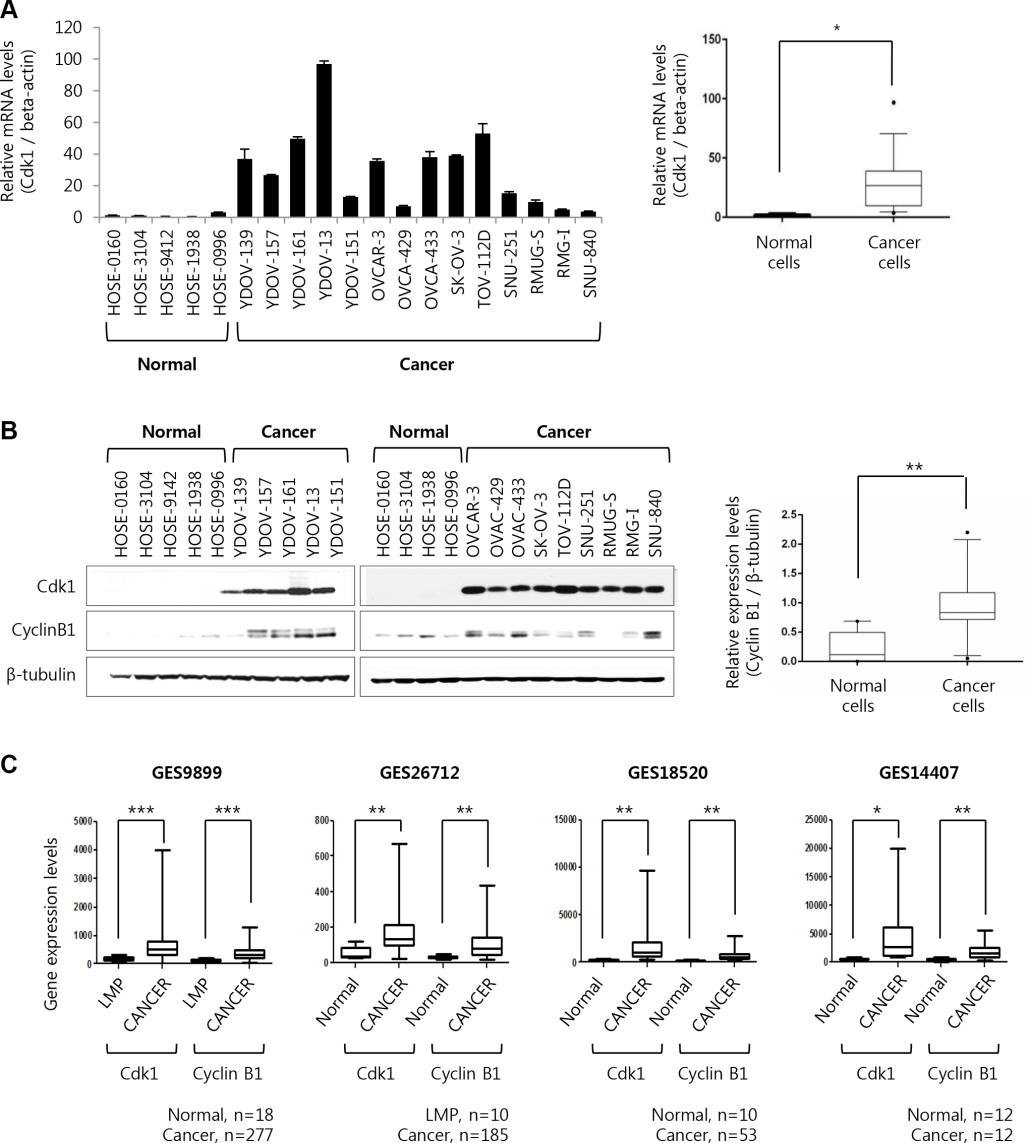
The expression levels of Cyclin dependent kinase 1 is induced in ovarian cancer cell lines and four Gene Expression Omnibus dataset (**A**) Five human normal ovarian epithelial cell and fourteen ovarian cancer cell lines were extracted at 70% cell density. Cdk1 mRNA levels were measured by real-time PCR. The fold-change is expressed as a ratio of beta-actin. Results are the means ± S.E. *n* = 3 (left panel). Box plot is corresponded to cdk1 mRNA expression depending on cell types as normal cells or cancer cells. *p < 0.05 (right panel). (**B**) The expression level of Cdk1, Cyclin B1, and β-tubulin were examined via Western blot analyses (left panel). Box plot is corresponded to Cyclin B1 protein expression depending on cell types as normal cells or cancer cells. The cyclin B1 band intensities in western blot were quantified as a ratio of β-tubulin band using the Image J 1.48v software (right panel). **p < 0.01. (**C**) mRNA expression level of Cdk1 and cyclin B1 were analyzed at ovarian cancer patients in four Gene Expression Omnibus (GEO) database (GEO accession number; GES9899, GES26712, GES18520, GES14407). *P*-value compared normal vs cancer or LMP vs cancer. Normal = Human ovarian surface epithelial tissue or cell, Cancer = Ovarian cancer tissue, LMP = Low malignant potential. *p < 0.05; **p < 0.01; ***p < 0.001.

Therefore, Cdk1 mRNA level was tested in all of EOC cell lines that had been maintained in the laboratory, which found that Cdk1 mRNA level was higher in EOC cell lines than in HOSE cells (Figure 2A). Protein expression level of Cdk1 was also higher in EOC cell lines, consistent with mRNA level (Figure 2B). In addition, a cyclinB1 as a Cdk1 binding partner, also increased in EOC cell lines as per Cdk1 expression (Figure 2B). Like the preceding in Figure 1, these results indicate that both of Cdk1′s expression and activity were increased in epithelial ovarian cancer cell lines. Subsequently, Cdk1 and cyclinB1 expression levels were analyzed in four gene expression profiling datasets (GES9899, GES26712, GES18520, and GES14407) in the Gene Expression Omnibus (GEO). These results showed that Cdk1 and cyclinB1 expression levels in four microarray datasets were higher in ovarian epithelial cancer tissues than in HOSE or LMP tissues, consistent with our results of microarray [16–18] and cell lines (Figure 2C). Therefore, these results indicate that expression levels of Cdk1 with its kinase activity is increased in ovarian cancer, and Cdk1 expression might significantly affect ovarian cancer cell growth, considering that the intrinsic function of Cdk1 is regulation of the cell cycle.

### Knockdown of Cdk1 by si-Cdk1 diminished the cell growth in ovarian cancer cell lines

**Figure 3:**
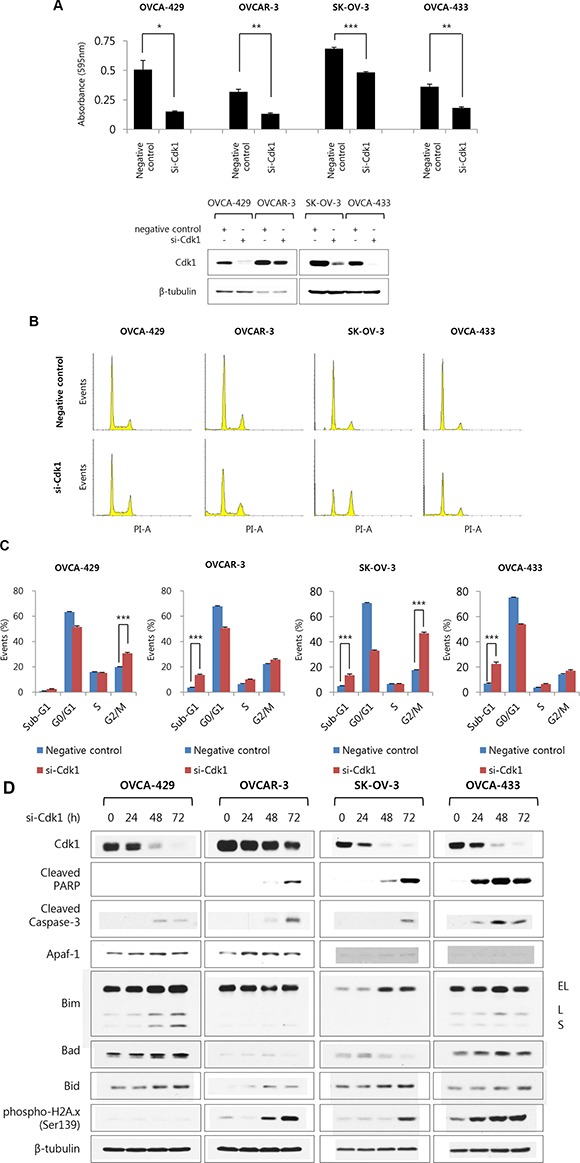
The si-Cdk1 inhibit cell growth via apoptosis and G2/M arrest in ovarian cancer cell lines (**A**) Four ovarian cancer cell lines (OVCA-429, OVCAR-3, SK-OV-3 and OVCA-433 cells) were transiently transfected with si-Cdk1 and si-negative control for 72 h. Cell viability was measured via crystal violet assay. Data are expressed as the means ± S.E. *n* = 3 (upper panel). Protein expression levels of Cdk1 were measured by western blot analysis (bottom panel). (**B**) Four ovarian cancer cell lines were introduced with si-Cdk1 or si-negative control for 72 h. siRNA introduced cells were stained propidium iodide and the cell cycle populations were evaluated via FACS analysis. Shown are representative images of 4 separate experiments. (**C**) Quantification of B. Results are the means ± S.E. *n* = 4. ***p < 0.001 (**D**) Four ovarian cancer cell lines was transfected with si-Cdk1 in indicated time-dependent manner. The expression level of indicated protein and the phosphorylation form of H2A.x was examined via Western blot analyses.

To determine whether elevated Cdk1 protein expression affects ovarian cancer growth, Cdk1 expression was inhibited by si-Cdk1. First of all, the effects of three different sequence of si-Cdk1 were examined in OVCAR-3 cells. Si-Cdk1#1 could not reduce Cdk1 expression. Si-Cdk1#2 and #3 decreased Cdk1 expression and caused apoptosis and G2/M arrest (Supplementary Figure 4). We selected si-Cdk1#2, which showed the strongest apoptotic effects, and observed its effects on various ovarian cancer cell lines. Ovarian cancer cell lines (OVCA-429, OVCAR-3, SK-OV-3, and OVCA-433) were transfected with si-negative control and si-Cdk1 and were incubated for 72 h. The si-Cdk1 transfected group of cell lines showed lower rate of cell growth than the si-negative control transfected group, the reduction rate of cell growth being: OVCA-429 = 70.1%, OVCAR-3 = 58.4%, SK-OV-3 = 29.5%, and OVCA-433 = 49.8% (Figure 3A). When ovarian cancer cell lines were treated with si-Cdk1 for 72 h, the sub-G1 population of OVCAR-3, SK-OV-3, and OVCA-433 cells was remarkably larger than that of si-negative control transfected cells. Additionally, OVCA-429 and SK-OV-3 cells were arrested at G2/M phase after transfection with si-Cdk1 (Figure 3B and 3C). Western blot analysis of the ovarian cancer cells shows an increase in apoptotic markers, such as cleaved PARP, cleaved caspase-3, Apaf-1, Bim, Bad, and Bid, in the si-Cdk1 transfected cells than in the si-negative control cells, in a time-dependent manner (Figure 3D). Previous report suggested that the knockdown of Cdk1 induced apoptosis in Myc-dependent human breast cancer cell [20]. In addition, although the overactivity of E2F1 increased cell death in cancer cells [21], the expression of E2F1 is increased and was correlated with FIGO stage, tumor grade in ovarian cancer [22]. Cdk1 increased phosphorylation of E2F1 for inhibitory effects of apoptotic function. During knock-down of Cdk1 expression, the overactivity of E2F1 increased cell death in cancer cells [21, 23]. Thus, these results suggest that the knockdown of Cdk1 could be induced apoptosis and cell death in ovarian cancer cells.

Moreover, phosphorylation of H2A.x (Ser139), which is a DNA-damage marker, increased in OVCAR-3, SK-OV-3 and OVCA-433 cells. Thus, it seems that DNA damage was induced by a reduction in Cdk1 expression (Figure 3D). In addition, expression levels of phospho-BRCA1 (Ser1524) and BRCA1 as a key regulated protein in DNA double-strand repair mechanism and homologous recombination were diminished in si-Cdk1 transfected ovarian cancer cell lines, in a time-dependent manner (Supplementary Figure 5A). According to these observations, ovarian cancer cells could be negatively affected by inhibition of Cdk1 expression, because of apoptosis and DNA damage.

### RO-3306 as a potent Cdk1 inhibitor inhibits cell growth in OVCA-429 and OVCAR-3 cell lines

**Figure 4:**
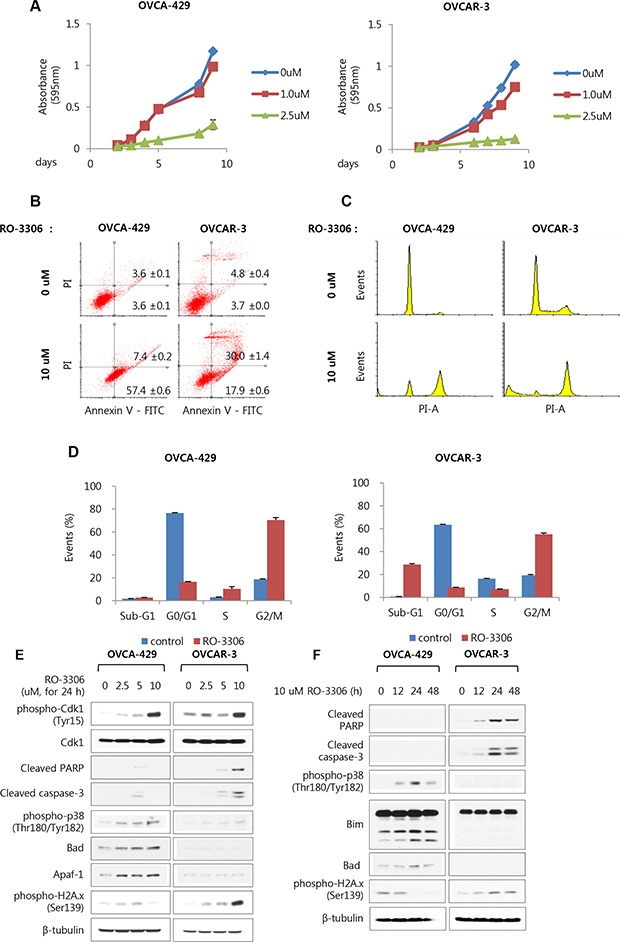
RO-3306 as a potent Cdk1 inhibitor inhibits cell growth via induction of apoptosis and G2/M arrest in OVCA-429 and OVCAR-3 cells (**A**) OVCA-429 and OVCAR-3 were exposed to RO-3306 in indicated time- and dose-dependent manner. Cell viability were measured via crystal violet assay. Results are the means ± S.E. *n* = 3. (**B**) OVCA-429 and OVCAR-3 were treated with 10 uM RO-3306 for 48 h. Detection of apoptosis with annexin V-FITC and propidium iodide (PI) via FACS analysis. (Dot plots, Left bottom side is live cells as both annexin V and PI negative. Right bottom side is early apoptosis cells as annexin V positive while still excluding PI. Right top side is late apoptosis cells as double positive of annexin V and PI.) (**C**) Under same condition, cell cycle were measured with PI staining via FACS analysis. Shown are representative images of 3 separate experiments. (**D**) Quantification of C. Results are the means ± S.E. *n* = 3. (**E**) OVCA-429 and OVCAR-3 were exposed to RO-3306 for 24 h in indicated dose dependent manner, (**F**) 10 uM RO-3306 for 12 h up to 48 h. The expression level of the indicated protein and the phosphorylated form of H2A.x and p38 were examined via Western blot analyses.

To confirm whether RO-3306, a potent Cdk1 inhibitor, also inhibits growth of ovarian cancer cells, OVCA-429 and OVCAR-3 cells were treated with RO-3306 at various concentrations. It was observed that the growth rates of OVCA-429 and OVCAR-3 cells were lower at high dose concentration of RO-3306 for up to 9 days. The growth rate of OVCA-429 and OVCAR-3 cells showed 75.3% and 87.7% reductions, respectively with 2.5 μM RO-3306 on the 9th day (Figure 4A). To investigate whether the decline in the cell growth was caused by cell cycle arrest or apoptosis, FACS analysis was performed with annexin V and propidium iodide (PI) staining. OVCA-429 and OVCAR-3 cells were treated with RO-3306 for 48 h, followed by staining with annexin V and PI, and analyzed using FACS. The proportion of the OVCAR-3 cells in late apoptosis increased from 4.8 to 30.0% with 10 μM RO-3306, while that of the cells in early apoptosis increased from 3.7 to 17.9%. The proportion of OVCA-429 cells in late apoptosis slightly increased from 3.6 to 7.4%, and that of cells in early apoptosis increased considerably from 3.6 to 57.4%. Thus, there is a slight difference between OVCA-429 and OVCAR-3. (Figure 4B). In the cell cycle analysis with PI staining, the RO-3306-treated OVCAR-3 cells showed an increase in the sub-G1 (0.8% at 0 μM to 28.7% at 10 μM) and G2/M population (19.3% at 0 μM to 55.1% at 10 μM). In case of OVCA-429 cells, the G2/M phase was arrested with RO-3306 treatment (18.6% at 0 μM to 70.4% at 10 μM), but the sub-G1 population was unchanged (Figure 4C and 4D).

Western blot analysis was performed to verify whether there were any changes in the apoptotic markers with treatment of RO-3306. After treating with RO-3306 for 24 h, the inhibitory phosphorylation of Cdk1 increased dose-dependently both in OVCA-429 and OVCAR-3 cells (Figure 4E). The levels of apoptotic marker proteins, cleaved PARP and cleaved caspase-3, increased both dose- and time-dependently with RO-3306 in OVCAR-3 cells (Figure 4E and 4F). However, in OVCA-429 cells which were treated with 5 μM RO-3306 for 24 hours, the levels of these proteins showed only slight increases (Figure 4E). Instead, phosphorylation of p38, a stress-dependent kinase, and the levels of apoptotic marker proteins Bad, Apaf-1, and Bim increased (Figure 4E and 4F). We also confirmed that phosphorylation of H2Ax, a DNA damage marker, increased in OVCAR-3 cells treated with RO-3306 in a dose- and time-dependent manner (Figure 4E and 4F). Moreover, phosphorylation of BRCA1, which is involved in double-strand break repair, and its reduced expression were observed 12 hours after treatment with RO-3306, after which both recovered gradually (Supplementary Figure 5B). These results indicated that the activity and expression of BRCA1 were partially reduced by Cdk1 inhibition. Accordingly, increased DNA damage could be observed.

Finally, these results indicate that inhibition of Cdk1 activity negatively affected growth of OVCA-429 and OVCAR-3 cells through distinct molecular mechanisms, consistent with transfection of si-Cdk1.

### Combined treatment with RO-3306 and cisplatin effectively induce apoptosis in OVCA-429 and OVCAR-3 cells

**Figure 5:**
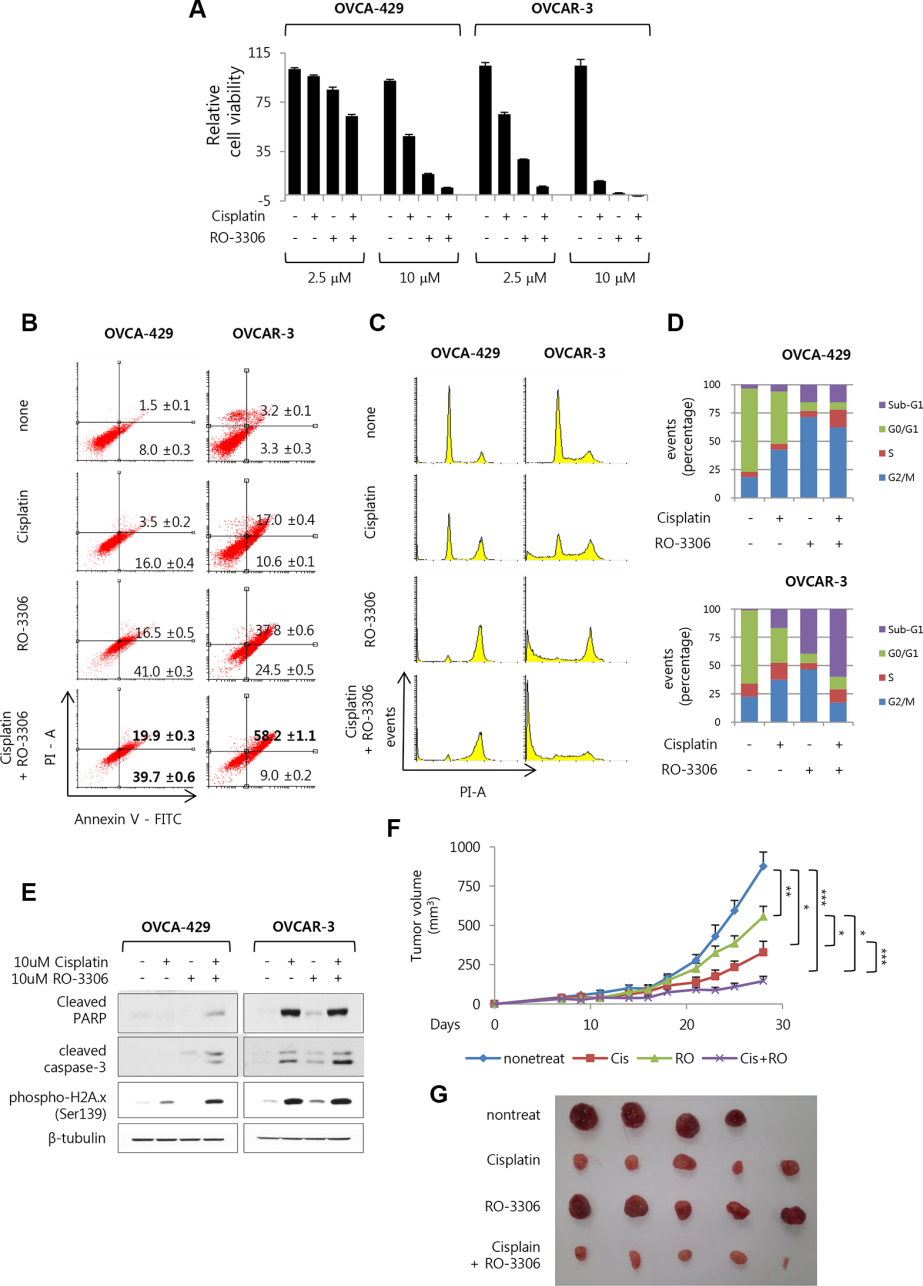
The combined treatment of RO-3306 and cisplatin effectively induces apoptosis in ovarian cancer (**A**) OVCA-492 and OVCAR-3 were exposed to RO-3306 in the presence of cisplatin for 5 days with combined treatment at 2.5 uM and 10 uM. Then cell viability was measured via crystal violet assay. Results are the means ± S.E. *n* = 3. (**B**) Under 10 uM concentration of drug treatment for 3 days, detection of apoptosis with annexin V-FITC and propidium iodide (PI) via FACS analysis in both of cell. (Dot plots, Left bottom side is live cells as both annexin V and PI negative. Right bottom side is early apoptosis cells as annexin V positive while still excluding PI. Right top side is late apoptosis cells as double positive of annexin V and PI.). (**C**) Cell cycle were measured with propidium iodide (PI) staining via FACS analysis. Shown are representative images of 3 separate experiments. (**D**) Quantification of C. Results are the means (*n* = 3). (**E**) OVCA-492 and OVCAR-3 were exposed to RO-3306 in the presence of cisplatin for 48 h. The indicated protein levels were measured via Western blot analysis. (**F**) BALB/c nude mice were inoculated subcutaneously into one flank with 3 × 10^6^ OVCAR-3. Control group were four mice and other group were five mice. Drugs were intraperitoneally administered once every 4 days. First day of drug injection is seventh day after cell inoculation (Cisplatin, 4 mg / kg; RO-3306, 4 mg / kg), (*p < 0.05; **p < 0.01; ***p < 0.001). (**G**) Gross images of tumor masses from xenograft mice from each group.

To investigate whether the inhibitory effect of RO-3306 on cell growth increased on combined treatment with cisplatin, OVCA-429 and OVCAR-3 cells were treated with either RO-3306 and cisplatin (2.5 μM and 10 μM), or individually with each drug. Cell viability was measured after 5 days using crystal violet assay. It was seen that 2.5 μM and 10 μM of the combined treatment effectively inhibited the growth of OVCAR-3 and OVCA-429 cells, respectively (Figure 5A). To accurately measure the rate of apoptosis following combined treatment with RO-3306 and cisplatin, FACS analysis was performed after staining with annexin V and PI. The result showed that late-apoptotic population in OVCA-429 cells increased to 19.9% following 10 μM combined treatment, in contrast to 3.5 and 16.5% following 10 μM cisplatin and RO-3306 individual treatments, respectively. On the other hand, the late-apoptotic population in OVCAR-3 cells increased considerably to 58.2% follwing 10 μM combined treatment (Figure 5B). Analysis of the cell cycle using PI staining revealed that subjecting the OVCA-429 cells to 10 μM combined treatment remarkably increased the population of sub-G1 and G2/M phase cells. In contrast, OVCAR-3 cells showed a greater sub-G1 population following a 10 μM combined treatment (Figure 5C and 5D). Western blot revealed that the OVCA-429 cells subjected to combined treatment showed higher phosphorylation of H2A.x, as a DNA damage marker, than did the cells treated with a single agent. In addition, the level of cleaved PARP and cleaved caspase-3 also increased slightly. On the other hand, the level of cleaved caspase-3 was higher in OVCAR-3 cells subjected to combined treatment than in single-agent treated cells (Figure 5E). To determine whether the combined treatment with RO-3306 and cisplatin had any effect on *in vivo* mouse model, subcutaneous xenograft of OVCAR-3 cells was performed in BALB/c nude mice. Consistent with the *in vitro* results, the xenograft model showed that the combined treatment effectively inhibited tumor growth, as opposed to treatment with either RO-3306 or cisplatin (Figure 5F and 5G). These results show that combined treatment with RO-3306, a Cdk1 inhibitor, and cisplatin effectively inhibits growth of OVCA-429 and OVCAR-3 cells.

## DISCUSSION

Previously, we performed microarray analysis with cell lines from one mucinous-type and one brenner tumor-type ovarian cancer, including three from high-grade serous type cancers that are representative histotypes of ovarian cancer [16–18]. Reanalysis of the microarray results revealed up-regulation of Cdk1 expression in the ovarian cancer cell lines. Furthermore, all 14 ovarian cancer cell lines maintained in our laboratory were found to have elevated levels of Cdk1 expression. The results in this study were similar to those published to date, which show higher Cdk1 expression and activity in Hodgkin's lymphomas, colorectal cancer, prostate cancer, gastric lymphoma, and childhood acute lymphoblastic leukemia [5–9]. This indicates that ovarian cancer also maintains a high level of Cdk1 expression and activity.

Above all, this study has an interesting point about Cdk1′s trait. There is whether cytoplasmic cdk1 which was elevated in the ovarian cancer can have activity, or not. It is well known that Cdk1 generally has its activity and regulates cell cycle in nucleus. But, we demonstrated that expression levels of cytoplasmic Cdk1 were increased in the results from ovarian cancer patient's tissue microarray (*n* = 249) and ovarian cancer cell lines (*n* = 14). Even there are large numbers of TMA slide cores which were stained cytoplasmic Cdk1 without nucleus Cdk1 staining in ovarian cancer (Figure 1 and Table 1). Also, Inhibitory phosphorylation form of Cdk1 (pTyr15) in epithelial ovarian cancer cell lines was increased in nucleus than in cytoplasm. Besides, cyclin B1 was also highly expressed in the cytoplasm of ovarian cancer cells. Cyclin A was expressed in the nucleus at high levels, but a certain level of expression was also observed in the cytoplasm (Figure 1F). In the past studies, Cdk1 in fly embryos can be activated in the cytoplasm independently from its activation in the nucleus [15]. Also, Cdk1-CycB complex is activated in the cytoplasm before its nuclear activation in mammalian cultured cells [14]. The expression of Cdk1 and CyclinB1 is higher in cytoplasm and mitochondria than in nucleus via subcellular fractionation in neurons and HEK293T cells [24]. When all is said and done, we imply that cytoplasmic Cdk1 expressed in ovarian cancer can be activated and this altered expression of cytoplasmic Cdk1 might influence in ovarian cancer growth. But, the precise mechanism whereby cytoplasmic Cdk1′s activity modulates ovarian cancer growth remains unknown. In addition, although the average cytoplasmic Cdk1 IHC score in ovarian cancer TMA slide was relatively low at 1.17, it was 3.44-fold higher than the average score of 0.34 in normal tissues (Figure 1C and Table 1). Moreover, considering the confirmed roles of Cdk1 from *in vitro* and *in vivo* experimental data (Figures 3, 5F, and 5G, and Supplementary Figure 4) and changes in patient prognosis according to Cdk1 expression (Figure 1D), our results hold clinical significance and can be used for clinical application.

Besides, Cdk1 is closely related with BRCA because BRCA2 is phosphorylated by Cdk1 and Cdk2 to induce DNA repair [25, 26]. And, Cdk1 directly induces the formation of BRCA1 foci during DNA damage response through phosphorylation of a number of serine residues in BRCA1 [25]. As per TCGA results, although 33% of ovarian cancer patients had alterations in BRCA, only a small proportion of patients showed mutations in both, BRCA1/2 [27]. In addition, although loss of heterozygosity was discovered in 41 ovarian cancer cell lines generally used in most laboratories, it was rare to find a complete loss of function caused by both of BRCA1 and BRCA2 mutations [28]. Most ovarian cancers retain the same amount of BRCA function as wild-type, regardless of the level of activity. In addition, the rate of recurrence was reportedly lower in patients with BRCA1/2 mutations in triple-negative breast cancer patients [29], and prognosis was poor when expression level of BRCA1 was high in sporadic epithelial ovarian cancer patients [30]. Thus, it is highly probable that since ovarian cancer shows high levels of Cdk1 expression and activity, ovarian cancer could exhibit resistance to DNA damage agents due to increased BRCA activity by Cdk1. Whereupon, it is also highly possible that recurrence and malignancy could be higher in ovarian cancer with higher levels of Cdk1 expression and activity, and prognosis of patients could be poor as a result.

Actually, when the expression of Cdk1 was reduced using si-Cdk1 or Cdk1′s activity was inhibited using RO-3306, as a potent Cdk1 inhibitor, DNA damage was increased in si-Cdk1 or RO-3306 treated cells (Figures 3 and 4). In at this point, the activity and expression of BRCA1 decreased in a time-dependent manner upon si-Cdk1 treatment. Upon RO-3306 treatment, the activity of BRCA1 was reduced initially and then recovered gradually with time (Supplementary Figure 5A and 5B). These results indicate that the inhibition of Cdk1 activity or expression can decrease BRCA1 activity, resulting in reduced cellular recovery from DNA damage. Further, the inhibition can potentially increase sensitivity of the cells to anticancer drugs. Actually, when ovarian cancer cell lines were treated with a combination of Cisplatin and RO-3306, as compared with either treatment alone, DNA damage increased (Figure 5E). However, although reduced expression of BRCA1 through inhibition of Cdk1 was observed, inhibitory effects on its activity were not (Supplementary Figure 5C). Although the decrease in BRCA1 activity upon Cdk1 inhibition was not prominent for combined treatment of RO-3360 and Cisplatin, it is certain that the inhibition of Cdk1 was significantly suppressed cell growth and promoted apoptosis of ovarian cancer cell lines treated with cisplatin. In same results, *in vivo* xenograft tumor was effectively inhibited via RO-3306 treated with cisplatin (Figure 5F and 5G).

As a result from this study, high expression of cytoplasmic Cdk1 could be a common characteristic of ovarian cancer, which is different from normal ovarian epithelial cells and be associated with patient's prognosis. Therefore, Cdk1 has potential as a desirable candidate gene for effective treatment of ovarian cancer and is expected to effectively inhibit ovarian cancer in combination with chemotherapeutic agents.

## MATERIALS AND METHODS

### Antibodies and reagents

Anti-Cdc2 (#9112), CyclinB1 (#4138), CyclinA (#4656), p38 (#9212), cleaved caspase-3 (#9664), Apaf-1 (#8723), Bim (#2933), Bad (#9239), Bid (#2002), Bax (#5023), cleaved PARP (#5625), and BRCA1 (#9010) antibodies were purchased from Cell Signaling Technology (Danvers, MA). Anti-Cdk1 (A17.1.1) was obtained from Thermo scientific (Rockford, IL). Anti-p53 (sc-126), p21 (sc-397), Lamin B (sc-6216) and β-tubulin (sc-9104) antibodies were obtained from Sante Cruz Biotechology (Santa cruz, CA). The phospho-specific of H2A.x (Ser139, #9718), Cdk1 (Tyr15, #9111), p38 (Thr180/Tyr182, #9211) and BRCA1 (Ser1524, #9009) antibodies were affinity purified (Cell Signaling Technology). RO-3306 as Cdk1 selective inhibitor was purchased from Tocris Bioscience (Bristol, United Kingdom), and cisplatin was obtained from Sigma Aldrich (St. Louis, MO). The stock solution of 5mM RO-3306 was dissolved in dimethyl sulfoxide (DMSO) and 1.66mM Cisplatin was dissolved in 0.9% sodium choloride.

### Cell culture

YDOV-13, YDOV-139, YDOV-151, YDOV-157, YDOV-161 and five human ovarian surface epithelial (HOSE) cells, which were established and characterized in our laboratory, were cultured as described previously [16–18]. SNU-251 and SNU-840 were purchased from Korea Cell Line Bank (KCLB, Seoul, Republic of Korea). RMUG-S and RMG-I were purchased from Japanese Collection of Research Bioresources Cell Bank (JCRB, Tokyo, Japan). OVCAR-3, SK-OV-3, and TOV-112D were purchased from American Type Culture Collection (ATCC, Manassas, VA). OVCA-429 and OVCA-433 were maintained in DMEM supplemented with 10% FBS with antibiotics. All purchased cell lines were maintained as recommended.

### Patients and tissue microarray

A total of 249 patients who are diagnosed with ovarian cancer at Gangnam Severance Hospital (Seoul, Republic of Korea) were enrolled in this study between 1993 and 2014. Before commencement of this study, approval was obtained from the Institutional Review Board of Gangnam Severance Hospital (IRB approval No. 3-2014-0267), and written consent to participate in the study was obtained from each person. Tissue microarray block sections (10 μm thickness) were deparaffinized in two changes of Histo-Clear^™^ II (National Diagnostics, Atlanta, Gerorgia), rehydrated in graded ethanol, and treated for 30 min with 3% H_2_O_2_ solution in methanol to block endogenous peroxidase. After blocking in 1% bovine serum albumin in TBS for 30 minutes, sections were incubated with rabbit polyclonal anti-Cdc2 antibody (#9116, Cell Signaling Technology) diluted to 1:50 for overnight at 4°C, followed by detection using Dako LSAB+ (Dako, Glostrup, Denmark). The reaction product was developed with 3,3′-diaminobenzidine chromogen solution (Dako). Sections were counterstained with hematoxylin and mounted in Faramount Aqueous Mounting Medium (Dako). Appropriate negative and positive controls were concurrently performed. Representative photomicrographs were recorded using a digital camera (Nikon, Tokyo, Japan). Negative controls were processed by omitting the primary antibody. Human testis tissue was used as a positive control for Cdk1 immunoreactivity. Staining for Cdk1 was scored as positive when cancer or normal tissue (Epithelium, inclusion cyst, and fallopian tube) showed cytoplasmic and nucleus immunoreactivity. Cdk1 staining results were scored based on staining intensity (0 = negative, 1 = weak, 2 = moderate, 3 = strong) and the percentage of positive cells (0 = 0%, 1 = 1 – 25%, 2 = 26 – 50%, 3 = 51 – 100% positive cells) (Supplementary Figure 1). The cut-off value of Cdk1 expression was determined using receiver operating characteristic (ROC) analysis. The sensitivity and specificity for discriminating death or alive was plotted at each IHC score and the cut-off value was established to be the point of the ROC curve where the sum of sensitivity and specificity was maximized [31] (Supplementary Figure 2). The overall score for each patient was further simplified by dichotomizing as either cdk1 low expression (overall score of < = 1) or cdk1 high expression (score of > = 2) based on 1.5 as the cutoff value. Slides were scored in the absence of any clinical data, and the final immunostaining score was the average score of three expert pathologists.

### Immunocytochemisty

Immunocytochemistry was performed on methanol-fixed OVCA-429, OVCAR-3 and SK-OV-3 cell lines using anti-Cdk1 antibody (Thermo Scientific, Rockford, IL) and anti-Cdc2 antibody (Cell Signaling Technology, Danvers, MA). Briefly, after seeding 5 × 10^5^ cells per well in Lab-Tek Chamber slide (Nunc, Rochester, NY), cell lines were treated with ice cold methanol for fixation. Then the endogenous peroxidase activity was quenched by 3% hydrogen peroxide solution for 10 min. Non-specific binding was prevented by incubation with 5% bovine serum albumin and 0.01% Triton X-100 with 1 × Phosphate buffer saline for 15 min. After that, the sections were incubated with anti-Cdk1 antibody (1:300 dilutions) and anti-Cdc2 antibody (1:630 dilutions) for overnight at 4°C. Antibody binding was detected using horseradish peroxidase-conjugated secondary antibody at 37°C for 30 min. Then sections were visualized by 3,3′-diaminobenzidine solution (DAKO, Seoul, Republic of Korea), counterstained lightly with hematoxylin (Sigma-aldrich) dehydrated with ethanol and observed using inverted microscopy (model CKX41, Olympus).

### Subcellular fractionation

Cytoplasm and nuclear fractions were prepared using ProteoJET^™^ Cytoplasmic and Nuclear protein Extraction kit (Fermentas, CA) according to the manufacturer's instructions. Briefly, the average ratio of protein extract between cytoplasmic fraction and nuclear fraction in the subcellular fractionation was 3.08. Thus, the whole cell lysate (15 ug), cytoplasmic (15 ug), and nuclear extract (5 ug) were separated by SDS-PAGE and transferred to a nitrocellulose membrane. Thereafter, expression of Cdk1 was detected using an anti-Cdk1 (Thermo Scientific's antibody) and an anti-Cdc2 (Cell Signal Technology's antibody) for cross-checking.

### Protein extraction and Western blotting

Total cell lysate were isolated using cell lysis buffer (150 mM NaCl, 50 mM Tris pH 7.4, 1% NP-40, 1 mM EDTA, 1 mM sodium orthovanadate, 1 mM NaF, 1 mM sodium pyrophosphate) containing proteinase inhibitor cocktail (Roche, Nutley, NJ). Cells were harvested on ice, lysates centrifuged, and protein concentrations determined with a BCA assay (Sigma-Aldrich). Samples were stored at −20°C. Proteins were separated by SDS-PAGE and electrophoretically transferred from gels to 0.2 um nitrocellulose membranes (Pall Corporation, Washington, NY). The protein bands were visualized using a chemiluminescence detection kit (Santa Cruz Biotechology) after binding with the HRP-conjugated secondary antibody.

### SYBR green real-time polymerase chain reaction

Total RNA was extracted from cells using TRIzol reagent, chloroform extraction and ethanol precipitation. From each sample, 0.8 μg RNA was reverse transcribed into cDNA by Maxima First Strand cDNA Synthesis Kits for RT-qPCR (Thermo Scientific, Wilmington, DE) according to the manufacturer's suggested protocol. SYBR Green real-time polymerase chain reactions (PCR) were performed using primer sets. The primers for the PCR analysis were as follows: for Cdk1 forward (5′-CATGGATTCTTCACTTGTTAAGGT-3′), Cdk1 reverse (5′-TCCACTTC TGGCCACACTTC-3′), β-actin forward (5′-ATTAAGGAGAAGCTGTGCTACGTC-3′), and β-actin reverse (5′-ATGATGGAGTTGAAGGTA GTTTCG-3′). Reactions were carried out on an ABI 7300 (Applied Biosystems, Foster City, CA) using TOPreal qPCR 2× PreMIX reagents (Enzynomics, Daejeon, Republic of Korea). Cycling conditions were 2 min at 50^°^C, 10 min at 95^°^C followed by 40 cycles of 15 sec at 95^°^C and 1 min at 60^°^C. The cytoskelectal gene β-actin was used to normalize the quantity of cDNA used for PCR. Relative messenger RNA expression was quantified using the comparative Ct (ΔCt) method and expressed as 2^−ΔΔCt^, where ΔΔCt = ΔE - ΔC, ΔE = Ct_E target_ – Ct_E β-actin_ and ΔC = Ct_c target_ – Ct_c β-actin_ (E is the experimental result and C is the control).

### siRNA transfection

The cells were seeded at 5 × 10^5^ cells / well in a 6 cm dish. Medium without antibiotics was added to each well so that the cells grew to 70% confluence, when the transfection was conducted. The 100 pmole siRNA transfection was prepared using Lipofectamine RNAiMAX Reagent (Life Technologies) according to the manufacturer's instructions. si-Cdk1#1, si-Cdk1#2, and AccuTarget negative control siRNA were purchased from BIONEER (Cat. 1028582, 1028584, and SN-1002, Seoul, Republic of Korea) and a custom sequence for si-Cdk1#3 was designed using Custom RNA service by BIONEER. The si-RNA sequences for knock-down of Cdk1 expression were as follows: for siCdk1#1 sense (5′-GAUGUGCUUAUGCAGGAUUdTdT-3′), siCdk1#1 anti-sense (5′-AAUCCUGCAUAAGCACAUC dTdT-3′), siCdk1#2 sense (5′-CCUGGUCAGUACAUGGA UUdTdT-3′), siCdk1#2 anti-sense (5′-AAUCCAUGUAC UGACCAGGdTdT-3′) siCdk1#3 sense (5′-GUGGAAUCU UUACAGGACUAUdTdT-3′), siCdk1#3 anti-sence (5′-AU AGUCCUGUAAAGAUUCCACdTdT-3′). After 72 hours, cell lysate was harvested for western blot analysis or suspended cell by trypsinization were obtained for FACS analysis.

### Cell viability measurement

Cells were seeded in a 24-well plate at a density of 1 × 10^5^ cells. Cells were transfected 100 μM si-Cdk1 per a well and were treated with RO-3306 and cisplatin for indicated periods. Cells were fixed using 10% acetic acid solution with 10% methanol and stained with 0.5% crystal violet for 1 hour, photographed and extracted using 1% SDS solution. Crystal violet extract from cells was measured absorbance at 595 nm using VERSA max^™^, microplate reader (Molecular Devices, Sunnyvale, CA). All experiments were performed in triplicate.

### FACS analysis

The binding of annexin V-FITC to externalized phosphatidylserine was used as a measurement of apoptotic cells with an Annexin V-FITC/Propidium iodide Apoptosis Kit (Biovision, Milpitas, CA) according to the manufacturer's instructions. Briefly, after si-Cdk1 transfection or treatment of RO-3306 with cisplatin in EOC cells for indicated periods, next, 0.1 ml of this cell suspension was transferred to a 15-ml tube and incubated with 5 ul of Annexin V-FITC and 5 ul of Propidium iodide for 15 min at 25°C in the dark. Finally, 0.4 ml of binding buffer was added, and samples were analyzed within 1 h by a FACScan flow cytometer (BD Biosciences, San Jose, CA). Samples were gated on the basis of forward versus side scatter for size, and the results are presented as the percentage of cells that were viable (Ann-V^−^ PI^−^), early apoptotic (Ann-V^+^ PI^−^), or late apoptosis (Ann-V^+^ PI^+^). And, total cells were harvested via trypsinization, collected by centrifugation, and washed in PBS for propidium iodide (PI) staining. The cells were subsequently fixed with 70% ethanol and resuspended in PBS containing 50 ug/ml PI and 0.1 mg/ml RNase A. After the viable cells were sorted, fluorescence intensity was measured with FACScan flow cytometer (BD Biosciences) using excitation and emission wavelengths of 488 and 620 nm, respectively.

### *In vivo* xenograft mouse model

Five-week old female BALB/c nude mice were injected with OVCAR-3 cells (3 × 10^6^ cells / mouse) in the right flank to form xenograft tumors. Four mice were used for control group and five mice were used for treated group. Intraperitoneal injections of RO-3306 and cisplatin at four-day intervals (4 mg/kg, seven injection per mice). Tumor dimensions were measured with digital calipers every two days and tumor volume was calculated according to the following formula: tumor volume (mm^3^) = length × width^2^ × 0.5. Tumor xenografts were harvested at 28 days after cell transplantation. All animal experiments were approved by the Institutional Animal Care and Use Committee at Yonsei University in Republic of Korea (IACUC Approval No. 2014-0321).

### Statistical analysis

Results are expressed as the means ± S.E. When test of homogeneity of variances was not assumed, the Mann-Whitney test or the Kruskal-Wallis test was used for continuous variables. Survival curves analysis was performed by the Kaplan-Meier method, and statistical significance was calculated by the log-rank test. All analyses were performed using Graphpad Prism 6 software. Differences were considered significant at a *p* value of < 0.05. (**p < 0.01; ***p < 0.001; ^#^*p*>0.05).

## SUPPLEMENTARY MATERIALS FIGURES AND TABLES



## Abbreviations

Cdk1: Cyclin-dependent kinase 1
GEO: Gene expression omnibus
TMA: Tissue microarray
FACS: Fluorescence-activated cell sorting
HOSE: Human ovarian surface epithelium
LMP: Low-malignant potential
NGS: Next-Generation Sequencing
IHC: Immunohistochemistry
TCGA: The Cancer Genome Atlas.
