# Supplementary material for: Accumulation of cytoplasmic Cdk1 is associated with cancer growth and survival rate in epithelial ovarian cancer
Source: Oncotarget. 2016 Jul 1;7(31):49481–97. doi: 10.18632/oncotarget.10373 (PMC5226523; doi:10.18632/oncotarget.10373)
Supplement: Supplementary file 1 [file oncotarget-07-49481-s001.pdf]

Accumulation of cytoplasmic Cdk1 is associated with cancer growth and survival rate in epithelial ovarian cancer

Supplementary Materials

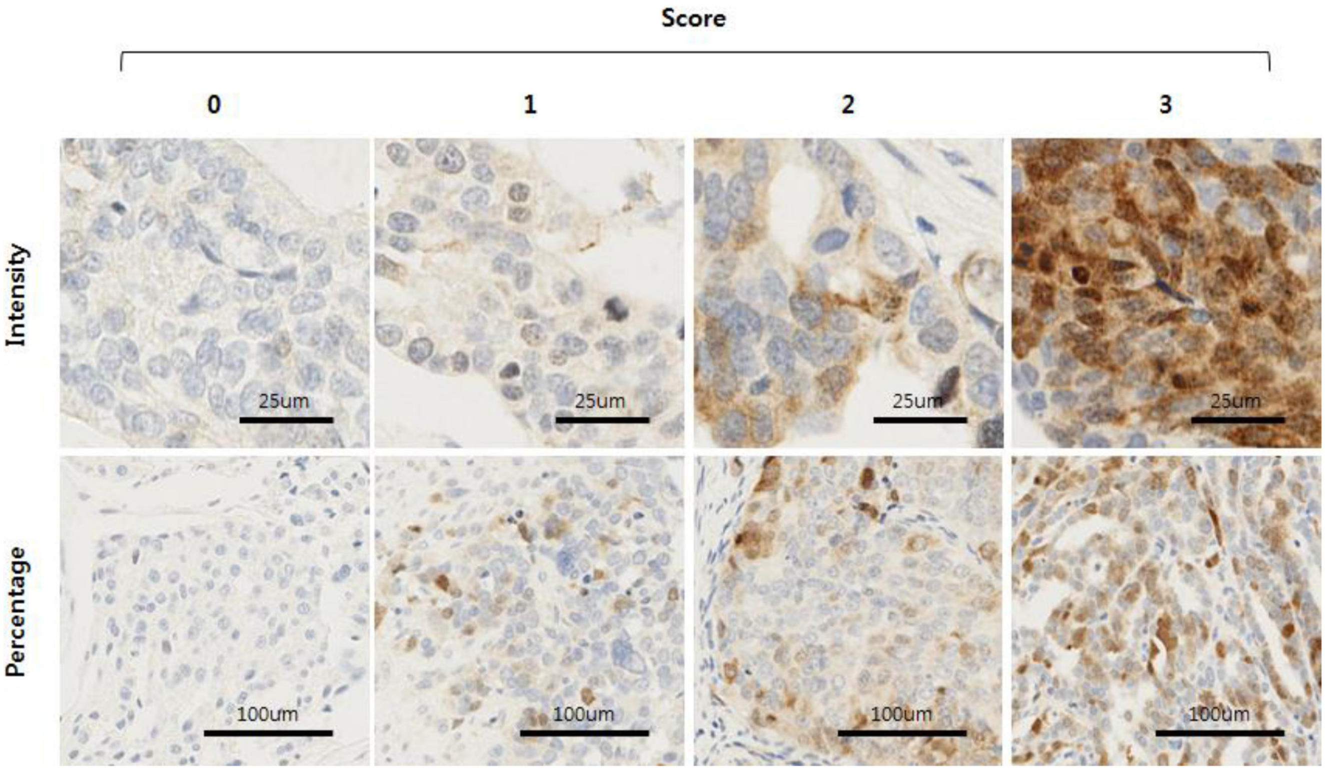

Supplementary Figure S1: Evaluation of Cdk1 IHC staining. Representative immuno-histochemical (IHC) staining images of the staining intensity (0 = negative, 1 = weak, 2 = moderate, 3 = strong) and the percentage (0 = 0%, 1 = 1 – 25%, 2 = 26 – 50%, 3 = 51 – 100% positive cells) of positive stained tissue.

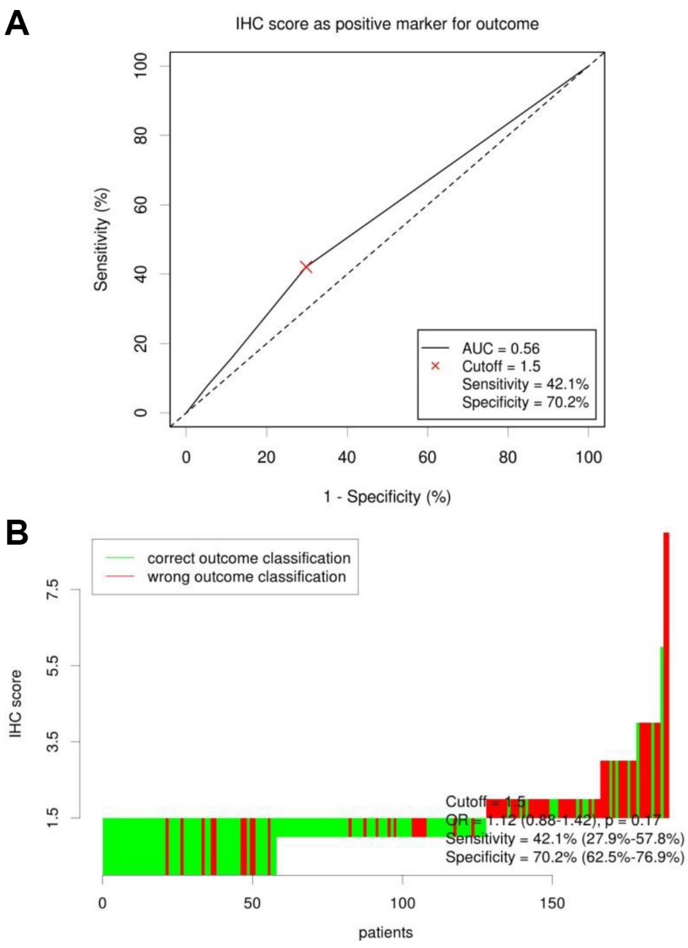

Supplementary Figure S2: The Optimal dichotomization of Cdk1 IHC staining in ovarian cancer. (**A**) Receiver operating characteristic (ROC) analysis was used when determining the cut-off point of Cdk1 IHC scores. Cut-off value was determined to be the point of the ROC curve where the sum of sensitivity and specificity was maximized. (**B**) The waterfall plot compared determination of immunohistochemical score according to status of Cdk1 expression using cut-off value from ROC curve.

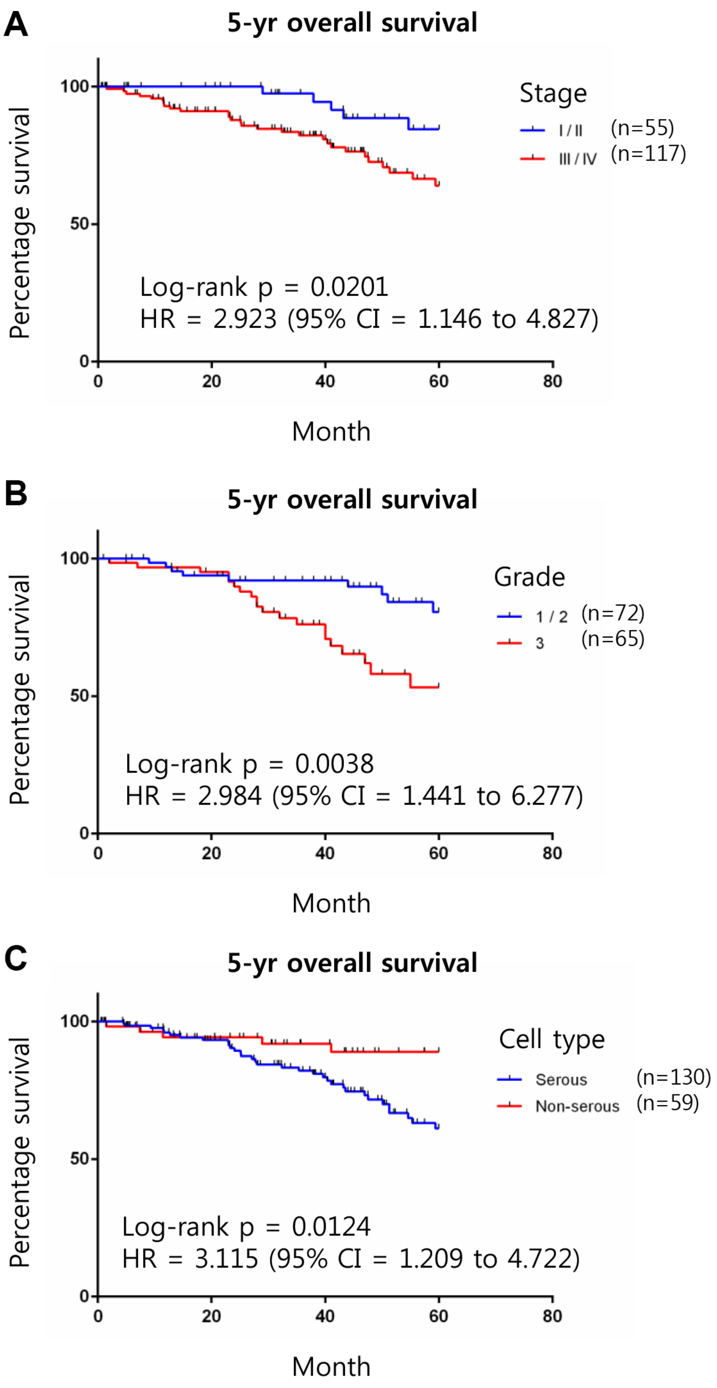

Supplementary Figure S3: Patients survival analyses. (A–C) Kaplan-Meier plots for patients with epithelial ovarian cancer were stratified according to FIGO stage (A), tumor grade (B), or cell type (Serous vs. Non-serous) (C).

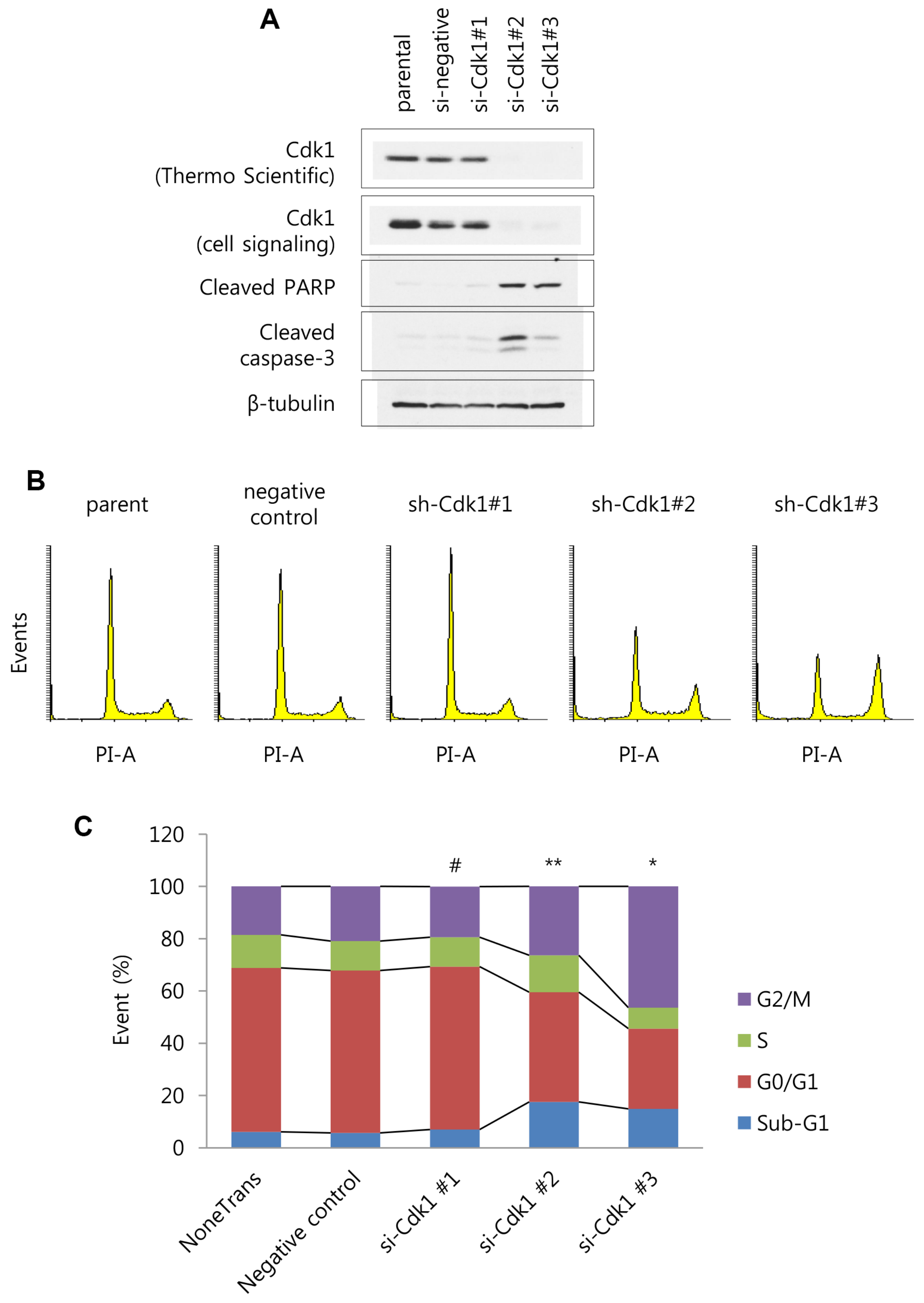

Supplementary Figure S4: The evaluation of three si-Cdk1 effects in OVCAR-3 cell lines. (**A**) OVCAR-3 cell lines were introduced with three si-Cdk1 which is different anti-sense sequence against Cdk1 mRNA each other and incubated for 72 h. The expression level of Cdk1, cleaved PARP and cleaved caspase-3 were examined via Western blot analyses. (**B**) Under same conditions, detection of cell cycle status with staining propidium iodide via FACS analysis. (**C**) Quantificaiton of B. Results are the means ± S.E. $n = 4$. (**strong apoptosis; *moderate apoptosis; $^{\#}$not effects).

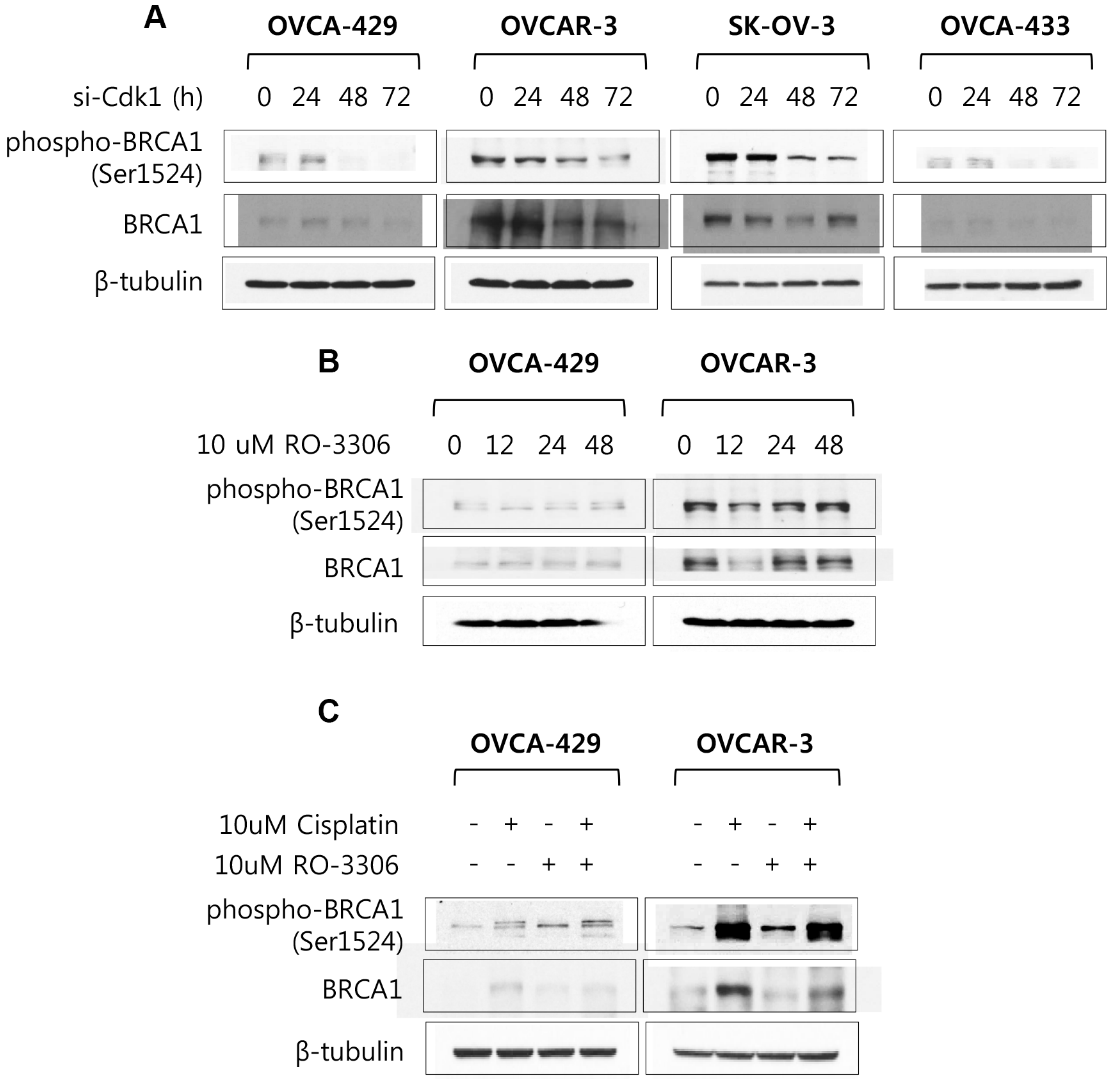

Supplementary Figure S5: Inhibition of Cdk1 using si-Cdk1 or RO-3306 supressed BRCA1 expression and activity. (**A**) Ovarian cancer cell lines (OVCA-429, OVCAR-3, SK-OV-3 and OVCA-433 cells) were transiently transfected with si-Cdk1#2 in time-dependent manner. (**B**) OVCA-429 and OVCAR-3 were exposed with 10 uM RO-3306 in indicated time periods. (**C**) OVCA-429 and OVCAR-3 were treated with RO-3306 in the presence of cisplatin for 48 h. The expression level of phospho-BRCA1 (Ser1524), BRCA1 and β-tubulin were measured by Western blot analyses.
